# Plasma metabolomics and gene regulatory networks analysis reveal the role of nonstructural SARS-CoV-2 viral proteins in metabolic dysregulation in COVID-19 patients

**DOI:** 10.1038/s41598-022-24170-0

**Published:** 2022-11-20

**Authors:** V. A. Ivanisenko, E. V. Gaisler, N. V. Basov, A. D. Rogachev, S. V. Cheresiz, T. V. Ivanisenko, P. S. Demenkov, E. L. Mishchenko, O. P. Khripko, Yu. I. Khripko, S. M. Voevoda, T. N. Karpenko, A. J. Velichko, M. I. Voevoda, N. A. Kolchanov, A. G. Pokrovsky

**Affiliations:** 1 Novosibirsk State University, Pirogova Str., 2, 630090 Novosibirsk, Russia; 2 Institute of Cytology and Genetics of Siberian Branch of Russian Academy of Sciences, Acad. Lavrentiev Ave., 10, 630090 Novosibirsk, Russia; 3 Kurchatov Genomics Center of Institute of Cytology and Genetics of Siberian Branch of Russian Academy of Sciences, Acad. Lavrentiev Ave., 10, 630090 Novosibirsk, Russia; 4 N. N. Vorozhtsov, Novosibirsk Institute of Organic Chemistry, Acad. Lavrentiev Ave., 9, 630090 Novosibirsk, Russia; 5 Federal Research Center for Fundamental and Translational Medicine, Timakov Str., 2, 630117 Novosibirsk, Russia; 6 Research Institute of Internal and Preventive Medicine–Branch of the Institute of Cytology and Genetics, Acad. Lavrentiev Ave., 10, 630090 Novosibirsk, Russia; 7 State Budgetary Healthcare Institution of Novosibirsk Region ‘City Clinical Hospital No. 11’, Tankistov str., 23, 630120, Novosibirsk, Russia

**Keywords:** Gene regulatory networks, Viral infection, Metabolomics

## Abstract

Metabolomic analysis of blood plasma samples from COVID-19 patients is a promising approach allowing for the evaluation of disease progression. We performed the metabolomic analysis of plasma samples of 30 COVID-19 patients and the 19 controls using the high-performance liquid chromatography (HPLC) coupled with tandem mass spectrometric detection (LC–MS/MS). In our analysis, we identified 103 metabolites enriched in KEGG metabolic pathways such as amino acid metabolism and the biosynthesis of aminoacyl-tRNAs, which differed significantly between the COVID-19 patients and the controls. Using ANDSystem software, we performed the reconstruction of gene networks describing the potential genetic regulation of metabolic pathways perturbed in COVID-19 patients by SARS-CoV-2 proteins. The nonstructural proteins of SARS-CoV-2 (orf8 and nsp5) and structural protein E were involved in the greater number of regulatory pathways. The reconstructed gene networks suggest the hypotheses on the molecular mechanisms of virus-host interactions in COVID-19 pathology and provide a basis for the further experimental and computer studies of the regulation of metabolic pathways by SARS-CoV-2 proteins. Our metabolomic analysis suggests the need for nonstructural protein-based vaccines and the control strategy to reduce the disease progression of COVID-19.

## Introduction

Coronaviruses (CoVs) represent a group of enveloped viruses with exceptionally long (26–32 kb) single strand positive sense ( +) RNA genome^1^. Human coronaviruses primarily affect the respiratory tract, causing fever, cough, and, in severe cases, the shortness of breath and systemic inflammation, possibly, resulting in sepsis, cardiac insufficiency and polyorganic dysfunction in high-risk patients. The pediatric problem of common cold coronaviruses (ccCoVs) discussed in the publication^2^ deserves a special interest. The virus causing COVID-19 (2019-nCoV or SARS-CoV-2) is more contagious, than previously identified human beta-coronaviruses (Severe Acute Respiratory Syndrome CoV/SARS-CoV and Middle East Respiratory Syndrome CoV/MERS-CoV). The origin of new SARS-CoV-2 variants due to the virus genome mutability is associated with the acquisition of stronger contagiosity and the ability to escape the immune control, which accounts to the faster spread of the infection^3^.

The 5` regions of coronaviral genome include the cap, 5` untranslated region (5’ UTR) and a long replicase gene comprising about 2/3 of the genome and coding for 16 non-structural proteins (nsp). The 3` genomic regions code for several structural proteins (spike (S), surface (E), membrane (M), and nucleocapsid (N) proteins) and a number of accessory proteins, which is variable in different coronaviruses. They also comprise the 3`-UTR and poly(A) tract^4^.

The replicase gene consists of two overlapping ORFs (ORF1a and ORF1b) coding for the components of viral replication and transcription complex (RTC). Replicase gene expression results in the translation of pp1a polyprotein from ORF1a, while the -1 ribosomal frameshift before the ORF1a translation termination codon causes a switch to ORF1b translation and production of pp1ab polyprotein representing a longer variant of pp1a. Cleavage by two viral cysteine proteases (papain-like PLpro and 3C-like 3Clpro) produces the intermediate precursor proteins and the highly conservative mature nonstructural protein, which can associate together to form the RTC^4^. The RTC is comprised of several enzymes, such as the nsp3 and nsp5 proteases, nsp7/nsp8 primase, nsp12 RNA-dependent RNA polymerase, nsp13 helicase/triphosphatase, nsp 14 exoribonuclease, nsp 15 endonuclease and nsp10/nsp16 N7- and 2`O-methyltransferase^5^. The 3` SARS-CoV genomic region codes for structural proteins and eight accessory proteins designated as ORF3a, 3b, 6, 7a, 7b, 8a, 8b and 9b. The SARS-CoV-2 3` genomic region codes for the same set of accessory proteins, with the exception of ORF3b^6^. Functions have been attributed to several SARS-CoV accessory proteins: ORF3a and ORF8a trigger the apoptosis, ORF7a activates NF-kB cascade, ORF3b regulates the expression of several cytokines and chemokines, ORF6 downregulates IFN production, while ORF8b induces the cellular DNA synthesis^7^.

The molecular mechanisms of viral infections and the related pathological processes are actively studied using metabolomic^8,9^, proteomic^10^, and transcriptomic analyses^11^.

Metabolomics measures the profiles of metabolites, such as amino acids, organic acids, bioamines, acylcarnitines, glycerophospholipids, sphingolipids, sugars and other compounds. Plasma metabolomics analyses of COVID-19 patients are represented in recent publications^12–16^. The metabolomic profiles of critical COVID-19 patients of intensive care facilities were characterized by the dominating change of kynurenine and arginine content (and arginine to kynurenine ratio), sarcosine and lysophosphatydilcholines. The creatinine and arginine to kynurenine ratio were the perfectly accurate predictors of mortality^9^. The systematic metabolomic analysis of sera from COVID-19 patients revealed a significant decrease in > 100 metabolites, such as aminoacids and their derivatives, compared to the control subjects^8^.

An investigation into COVID-19-associated processes and pathways is important for the identification of diagnostic and prognostic biomarkers and improvement of COVID-19 treatments. The data on the interactions between SARS-CoV-2 and human proteins laid the background for the study of molecular mechanisms of virus-host relationship^17^. Identification of the involvement of particular viral proteins in signaling pathways, via which these proteins affect the host biological functions, is a promising approach to the search of pharmacological targets of the new generation antivirals. However, while interpreting the experimental data, the role of viral proteins is often disregarded.

An establishment of the links between the different omics data and the viral proteins requires the use of gene network reconstruction methods. Previously, we developed the ANDSystem software and information system for gene network reconstruction based on the information extracted from factual databases or obtained by text-mining of research publications^18–20^. In particular, the reconstruction of pre-eclampsia associome^21^, identification of a novel tuberculosis susceptibility candidate^22^, reconstruction and analysis of the HCV interactome^23^, the search for novel candidate genes important for asthma and hypertension comorbidity^24^, the analysis of programmed cell death upon SARS-CoV-2 infection^25^, etc., has been performed with the use of ANDSystem.

In this study, we performed metabolomic analysis of blood plasma from COVID-19 patients and the control group using the high-performance liquid chromatography coupled with tandem mass spectrometric detection (LC–MS/MS). The proposed approach to metabolomic analysis has been previously employed by ourselves for the study of metabolomic profiles of cerebrospinal fluid and blood plasma samples of high-grade glioma patients^26^.

We identified 103 metabolites, which significantly differ between the compared groups. The highest level of significance was demonstrated by mevalonolactone (a cyclic form of mevalonic acid), which plays a key role in cholesterol biosynthesis. The content of this metabolite in the plasma of COVID-19 patients was higher than that in the control samples. The role of cholesterol biosynthesis in the mechanisms of COVID-19 pathologies including the cytokine storm drags the attention of many researchers^27^.

The amino acid metabolism and the biosynthesis of aminoacyl-tRNAs represented KEGG metabolic pathways^28^ enriched with metabolites, which significantly differ between the COVID-19 patients and the controls. Employment of ANDSystem enabled us to reconstruct the gene networks describing the potential molecular mechanisms, by which the viral proteins affect metabolic pathways perturbed in COVID-19 patients. Some viral proteins had multiple ways via which they can regulate different metabolic processes. Three viral proteins were involved in the greater number of regulatory pathways, namely, ORF8, E and nsp5. Noteworthy, the reconstructed gene networks only provide the hypothesis on the molecular mechanisms of virus-host relationship in COVID-19 pathology, which require further corroboration by experimental studies and computer simulation.

## Materials and methods

### Study subjects

Study subjects were enrolled at the State budgetary healthcare institution of Novosibirsk region ‘City Clinical Hospital No. 11’ and included patients with COVID-19 diagnosis confirmed by PCR and healthy controls. General patient cohort characteristics are shown in Table 1.

**Table 1:** Patient characteristics in two cohorts showing matching in terms of age and gender.

Group	Age		Gender	
	Confidence limits, lower	Confidence limits, upper	Male	Female
Control	66.25	75.92	9	10
COVID-19	62.92	73.08	16	14

The samples of blood plasma from the biobank subjects obtained in 2019 (before the COVID-19 epidemics in Russia) and stratified for sex and age were used as the controls. No exclusion criteria have been applied to the subjects of this cohort.

### Compliance with ethical standards

The study was reviewed and all experimental protocols were approved by the Ethics Committee of Novosibirsk State University Zelman`s Institute of Medicine and Psychology (Meeting Minutes of 02.11.2020). All procedures involving human participants were found to be compliant with the ethical standards of the institutional research committee and the 1964 Helsinki Declaration and its subsequent amendments or similar ethical standards. An informed consent form was completed and signed by every study subject.

### Plasma collection

Plasma samples were collected from study subjects by venipuncture and blood collection into a vacutainer containing potassium EDTA as stabilizer. Blood cells were pelleted down by centrifugation, and plasma samples were aliquoted and kept frozen at − 80 °C until further use.

### Sample preparation

All samples were processed at the same time according to the protocol described by^29^. Briefly, 100 µL of plasma sample were precipitated with 400 µL of cooled methanol and incubated overnight at − 80 °C for protein precipitation. Then, samples were centrifuged at + 4 °C and 16,000 G for 15 min. Supernatant was transferred into a new polypropylene tube and dried in a SpeedVac concentrator centrifuge (Thermo Fisher Scientific/Savant, Waltham, MA). Samples were reconstituted in 100 µL of water/acetonitrile (95:5) and subjected to a modified targeted metabolomics analysis with relative quantification. Each sample was analyzed in three replicates.

### LC–MS/MS analysis

Samples were analyzed using a Shimadzu LC-20AD Prominence chromatograph (Shimadzu Corporation, Japan) equipped with SIL-20AC autosampler (Shimadzu Corporation, Japan) thermostated at 10 °C. Sample (10 μL) was injected onto a Prontosil 120–5-C18 AQ (2.1 × 75 mm) (Econova LLC, Russia). The mobile phases consisted of HPLC buffer A (water containing 0.1% formic acid) and HPLC eluent B (100% acetonitrile), the flow rate during analysis was 0.25 mL/min. The HPLC elution gradient was as follows: from 0 to 3 min, the mobile phase B was decreased from 97% B to 85%; from 3 to 4 min, the percentage of solvent B was decreased from 85 to 30%; from 4 to 10 min, the mobile phase B was decreased to 2% and was kept at 2% for additional 4.5 min. At minute 14.5, solvent B was increased back to 97% and the column was equilibrated for additional 2.5 min at the flow rate of 0.5 mL/min.

Metabolites (*n* = 289) were analyzed in MRM mode. Data acquisition was performed on API 6500 QTRAP mass-spectrometer (AB SCIEX, USA) equipped with an electrospray ionization source operating in the positive/negative switch mode. The main mass spectrometric parameters were as follows. The IS (ion spray) voltages were set at 5500 V and − 4500 V for positive and negative modes, respectively. The ion source temperature was set at 475 °C, CAD gas was set as “medium”, Gas1, Gas2 and curtain gas were 35, 35 and 30 psi, respectively. Declustering potential was at 93 V, entrance potential at 10 V, and collision cell exit potential at 20 V for positive and negative ion modes. In addition, the polarity switching (settling) time was set at 5 ms, and dwell time was 3 ms for each MRM transition. The precursor ion and fragment ion transitions, the metabolite names, dwell times, and the appropriate collision energies for both positive and negative ion modes were adapted from^29^ with several metabolite transitions added by our group. The device was controlled and information collected using Analyst 1.6.3 software (AB SCIEX).

### Statistical analysis

To get the significance of the difference between metabolite level means in COVID-19 patients and the controls, Welch’s t-test implemented in the SciPy package v1.8.0 was used^30^. The multiple hypothesis testing was performed using the Benjamini-Yekutieli procedure from the statsmodels Python package v0.13.2^31^ (https://www.statsmodels.org/stable/index.html.

### ANDSystem

ANDSystem^19^ includes the global gene network defining the interactions between the molecular genetic objects constructed via automatized mining of research literature and data extraction from factological databases. Overall, the ANDSystem considers 13 types of objects (proteins, genes, metabolites, etc.) and 24 types of interactions (physical interactions, expression regulation, activity regulation, stability regulation, etc.). ANDVisio program module provides the graphical interface offering the user`s access to the database, which enables one to search for the pathways in the global gene network using the templates. The templates represent the linear chain of objects and their interactions. Additionally, the objects can be defined by the concrete names/identifiers or by an object type, only. In the first case, the pathway search will consider only the identified objects, otherwise, all the objects of a particular type will be considered.

In an attempt to identify the potential molecular mechanisms, via which the viral proteins could perturb metabolic pathways, we reconstructed the pathways describing different types of interactions between molecular objects using ANDSystem, which employs the rules described by special predesigned templates.

The program searches for those pathways in the global network, which meet the template description. In this work, we analyzed 7 such templates (Table 2). The first position in every template is occupied by SARS-CoV-2 proteins, the last one, the KEGG metabolic pathway proteins. Viral proteins can participate in protein–protein interactions with human counterparts, only. The length of the template chain was 2 to 5 objects. Only proteins and genes were considered as the objects here. The interactions included the protein–protein interactions, the regulatory interactions, and the links between the gene expression and the gene product, or similar ones.

**Table 2:** Templates for virus-host interaction pathways.

Template name	Template description*
P_1_	Vp – *PPI* – > Kp
P_2_	Vp – *PPI* – > Hp – *PPI* – > Kp
P_3_	Vp – *PPI* – > Hp – *Act/Stab/Pr/PPM/Tr* – > Kp
P_4_	Vp – *PPI* – > Hp – *Exp reg* – > Kg – *Exp* – > Kp
P_5_	Vp – *PPI* – > Hp – *Expreg*– > Hg – *Exp*– > Hp– *Expreg*– > Kg – *Exp*– > Kp
P_6_	Vp – *PPI* – > Hp – *Exp reg* – > Hg – *Exp* – > Hp – *Act/Stab/Pr/PPM/Tr* – > Kp
P_7_	Vp – *PPI* – > Hp – *Expreg*– > Hg – *Exp*– > Hp – *PPI* – > Kp

Interactions: *PPI* protein–protein interactions, *Act/Stab/Pr/PPM/Tr* regulation of activity or stability, or proteolysis, or post translational modifications, or transport (release). *Exp reg* regulation of gene expression. *Exp* gene expression (protein production).

*Objects: *Vp* SARS-CoV2 proteins, *Kp* KEGG metabolic pathway proteins, *Kg* KEGG metabolic pathway genes, *Hp* any host proteins inv olved in the interactions, *Hg* any host genes involved in the interactions.

### Statistical significance of the virus-host signaling pathways

The statistical significance of the link between the virus-host signaling pathways and KEGG metabolic pathways^28^ (*p* value) was estimated for each pathway template (Table 2) using hyperheometric distribution. For the calculations, all human proteins from KEGG database (N = 1393) were taken. The signaling pathways that link the viral proteins to the KEGG proteins from the list (N) were searched for with the use of templates. The use of a single template may have resulted in the identification of a set of signaling pathways. Based on the analysis of these sets, for each template P_i_, the number of proteins n_i_ from the list (N), which represent the last object in, at least, a single signaling pathway from the set of pathways of a particular template, is calculated. Similarly, for each metabolic pathway j including K_j_ proteins, the number of proteins, k_ij_, which are the targets of virus-host signaling pathways of template P_i_, is calculated. The probability of observing k_ij_ number due to accidental causes is estimated with the standard hypergeometric disctribution using hypergeom function of SciPy 1.8.0 package (https://scipy.org).

## Results

The metabolite content in plasma samples of COVID-19 patient and control cohorts is shown in the Supplementary Data. The filtration of metabolites was first approached. Only those metabolites that showed the non-zero values in at least two of three repeats were used for the further analysis. Thus, only 140 metabolites out of 289 metabolites that can be detected by the employed method were selected. The comparison of mean metabolite content in plasma samples of COVID-19 patients and the controls showed the statistically significant differences (Benjamini-Yekutieli test, *p* < 0.05) for 103 out of 140 metabolites studied (Supplementary Table S1).

KEGG metabolic pathway overrepresentation analysis (Table 3) performed with MetaboAnalyst websystem^32^ enabled us to identify the pathways enriched with metabolites from the list of 103 significant ones (Supplementary Table S1). The numbers of significantly overrepresented pathways (*q* < 0.05) are 3 and 6, respectively, when Holm or FDR corrections for multiple comparisons are accounted for. All KEGG processes from this list were linked to the amino acid metabolism or the aminoacyl-tRNA biosynthesis.

**Table 3:** Analysis of KEGG metabolic pathway overrepresentation.

Metabolite set	Total	Hits	Expect	*p* value	*q* value,Holm P	*q* value, FDR
Aminoacyl-tRNA biosynthesis	48	14	2.93	3.33E-7	**2.8E-5**	2.8E-5
Glycine, serine and threonine metabolism	33	10	2.02	1.29E-5	**0.00107**	5.41E-4
Arginine biosynthesis	14	6	0.855	8.95E-5	**0.00734**	0.00251
Valine, leucine and isoleucine biosynthesis	8	4	0.489	7.58E-4	0.0614	0.0159
Arginine and proline metabolism	38	8	2.32	0.00153	0.123	0.0258
Histidinemetabolism	16	5	0.978	0.00195	0.154	0.0273
Pantothenate and CoA biosynthesis	19	5	1.16	0.00449	0.35	0.0538
Alanine, aspartate and glutamate metabolism	28	6	1.71	0.00558	0.429	0.0586
Phenylalanine, tyrosine and tryptophan biosynthesis	4	2	0.244	0.0204	1.0	0.191
Purine metabolism	65	8	3.97	0.0403	1.0	0.29
Pentose phosphate pathway	22	4	1.34	0.0409	1.0	0.29
Glyoxylate and dicarboxylate metabolism	32	5	1.96	0.0414	1.0	0.29

Significant values (*q* < 0.05) are in bold.

We estimated the statistical significance of associations between the virus-host signaling pathways and KEGG metabolic processes (Table 4). Three overrepresented metabolic pathways, for which HOLM *p* value is < 0.05 (Table 3), are analyzed here.

**Table 4:** Statistical significance of associations between virus-host signaling pathways and KEGG metabolic processes.

Virus-host pathway template (P_i_)	Aminoacyl-tRNA biosynthesis (K_1_ = 40 proteins)		Glycine, serine and threonine metabolism (K_2_ = 34 proteins)		Arginine biosynthesis (K_3_ = 18 proteins)	
	k_i1_	*P* value	k_i2_	*P* value	k_i3_	*P* value
P_1_	3	0.092	0	–	0	–
P_2_	19	**0.00011**	11	0.26	10	**0.0075**
P_3_	3	0.23	0	–	2	0.99
P_4_	0	–	1	0.69	3	**0.009**
P_5_	5	0.981	10	**0.049**	10	**0.004**
P_6_	4	0.712	3	0.78	5	**0.024**
P_7_	19	**0.0089**	10	0.54	11	**0.021**

Significant values (*p* < 0.05) are in bold.

K_j_ is the number of proteins in KEGG metabolic pathway j, k_ij_ is the number of proteins, which represent the targets of virus-host pathway template P_i_ (Table 2).

As one can see from (Table 4), five out of seven templates are significant for arginine biosynthesis. The regulation of a particular KEGG pathway by viral proteins can occur via different signaling pathways and can involve different types of interactions. Conversely, only template P_5_ is significant for glycine, serine and threonine metabolism pathway. The signaling pathways described by templates P_2_ and P_7_ can significantly contribute to the regulation of aminoacyl-tRNA biosynthesis. A typical feature of these pathways is that the last link in the chain of interactions is represented by protein–protein interaction involving KEGG enzyme. Such pathways affect the activity and stability of proteins via protein–protein interactions, but not the expression of KEGG pathway enzymes.

A detailed analysis of signaling pathways associated with each of KEGG metabolic processes is provided below.

### Aminoacyl-tRNA biosynthesis

The first column in the table of KEGG metabolic pathway overrepresentation analysis guided by the identified set of metabolites, which significantly differ between COVID-19 and control groups, is occupied by aminoacyl-tRNA biosynthesis (Table 3). Overall, 48 metabolites are involved in the process, 14 of which are on the list of significantly differing metabolites (Supplementary Table S2).

Of them, ten metabolites are significantly increased in plasma samples of COVID-19 patients (L-Aspartic acid, L-Serine, L-Glutamic acid, etc.), while L-Asparagine, L-Tyrosine, L-Methionine и L-Leucine/L-Isoleucine contents are reduced there.

The reconstruction of signaling pathways potentially involved in the regulation of aminoacyl-tRNA biosynthesis by viral proteins was performed for the proteins localized to the mitochondria or the cytoplasm, separately (Supplementary Table S3). Of seven types of pathways, those described by P_2_ and P_7_ templates (Table 4) are significant. As previously described, these templates share a common feature. In them, the effect on the last target in the pathway is exerted by protein–protein interactions. P_2_ pathways are shorter, since they include only a single intermediate. These pathways involve protein–protein interactions only.

**Figure 1:**
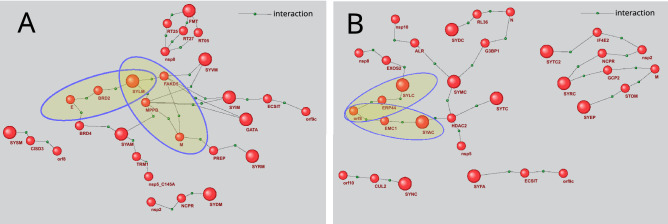
Gene networks describing mitochondrial (**A**) and cytoplasmic (**B**) P_2_ pathways, by which the virus, potentially, affects the proteins of aminoacyl-tRNA biosynthesis. The bigger balls show the proteins of aminoacyl-tRNA biosynthesis, while the smaller ones designate other proteins. The pathways discussed in the text are outlined.

Mitochondrial network comprising all signaling pathways identified with the use of P_2_ template includes 28 proteins and 31 interactions (Fig. 1A). The network contains 7 viral proteins and 9 enzymes of aminoacyl-tRNA biosynthesis. The cytoplasmic P_2_ pathways include 32 proteins, nine of which are viral proteins, while ten are the aminoacyl-tRNA biosynthesis enzymes (Fig. 1B). Merge of cytoplasmic and mitochondrial P2 networks results in 53 protein members (11 viral proteins and 19 aminoacyl-tRNA biosynthesis enzymes) and increases the number of interactions to 57.

**Figure 2:**
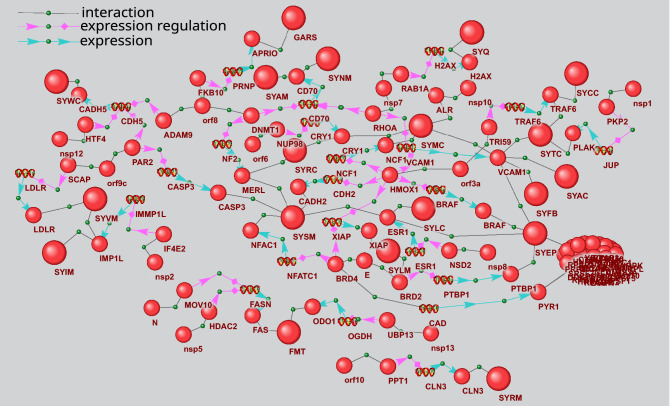
Gene networks describing P_7_ pathways, by which the virus, potentially, affects the proteins of aminoacyl-tRNA biosynthesis. The bigger balls show the proteins of aminoacyl-tRNA biosynthesis, while the smaller ones designate other proteins. Spirals designate the genes.

The merged P_7_ network of mitochondrial and cytoplasmic signaling pathways is shown in Fig. 2. P_7_ pathways include 3 intermediates (Table 2). The first intermediate is a protein interacting with viral protein. The expression of a gene, which represents the second intermediate, is regulated by intermediate 1. The third intermediate is the protein product of intermediate gene 2. At the end of pathway, intermediate 3 is involved in protein–protein interactions with a target protein of the considered KEGG metabolic process. As seen in Fig. 2, the P7 pathway network is greater than the P2 network. It includes 111 proteins (15 viral proteins and 19 aminoacyl-tRNA biosynthesis enzymes), as well as 23 genes. The network also involves 110 interactions, with 60 of them being protein–protein interactions, 27–the regulation of expression, and 23–the expression itself (production of protein gene products).

Noteworthy, the cytoplasmic and mitochondrial pathways of aminoacyl-tRNA biosynthesis regulation by viral proteins are different. For example, Fig. 1B shows that SYLC (cytoplasmic leucine-tRNA ligase) can be indirectly affected by 2 viral proteins, orf8 and nsp8. According to the recent publication^17^, orf8 interacts with Endoplasmic reticulum resident protein 44 (EPR44), which, in turn, can interact with SYLC (BioGrid Id 922,540). Functional effects of these interactions require further investigation. ERp44 supervises the correct assembly of multimeric proteins linked by disulfide bonds in the endoplasmic reticulum and their secretion^33^.

Exosome complex component RRP4 (EXOS2) can serve as an intermediate between Nsp8 and SYLC, as the information about EXOS2 and SYLC interaction is contained in BioGrid database (Id 2,457,178). In the cytoplasm, the RNA exosome complex is known to be involved in general mRNA turnover due to its specific degradation of inherently unstable mRNAs containing AU-rich elements in 3' untranslated regions and in RNA surveillance pathways, due to prevention of aberrant mRNA translation^34^.

According to Fig. 1A, the mitochondrial Leucine-tRNA ligase (SYLM) can be affected by viral proteins E and M. M protein can act onto SYLM via two pathways. One is mediated by MPPB (Mitochondrial-processing peptidase subunit beta), the other one by FAKD5 (FAST kinase domain-containing protein 5). MPPB cleaves the mitochondrial sequence off the newly imported precursor proteins^35^. BRD2 (Bromodomain-containing protein 2) turnes out to be an intermediate between E and SYLM. The data on MPPB, FAKD5 and BRD2 interactions with SYLM is contained in BioGrid (Id 2,857,559, Id 28,550,757 and Id 2,538,529, respectively).

Of note, all the discussed signaling pathways are only hypothetic and based on the integration of knowledge obtained from different experiments, thus, they require further corroboration.

### Glycine, serine and threonine metabolism

Glycine, serine and threonine metabolism is the second top process of overrepresented KEGG processes (Table 3). Of 33 metabolites participating in this process, ten are significantly different between plasma samples of COVID-19 patients and the controls (Supplementary Table S4). Nine of the latter are increased in content, while glyceric acid is decreased (logFC =  − 0.67).

**Figure 3:**
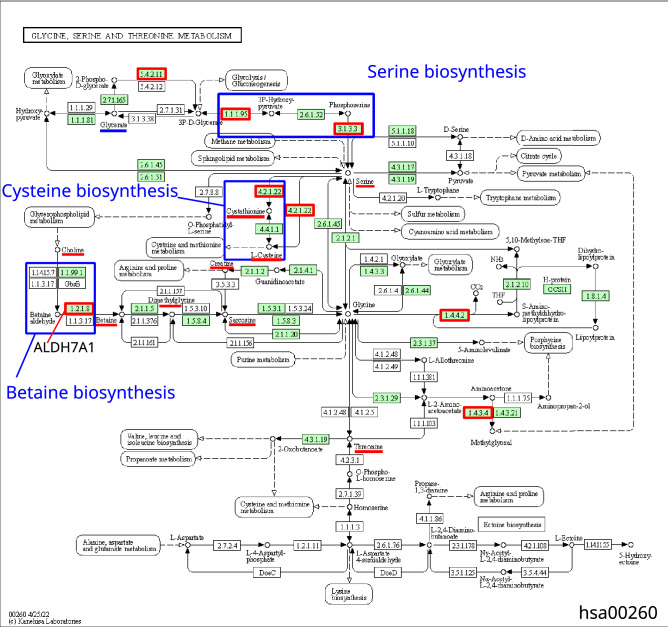
The scheme of glycine, serine and threonine metabolism extracted from KEGG database^28^ (https://www.kegg.jp/pathway/hsa00260). Enzymes that can be the potential targets of viral proteins are shown by red boxes, metabolites increased in the plasma of COVID-19 patients are underscored by red line, while those decreased are underscored by blue line.

**Figure 4:**
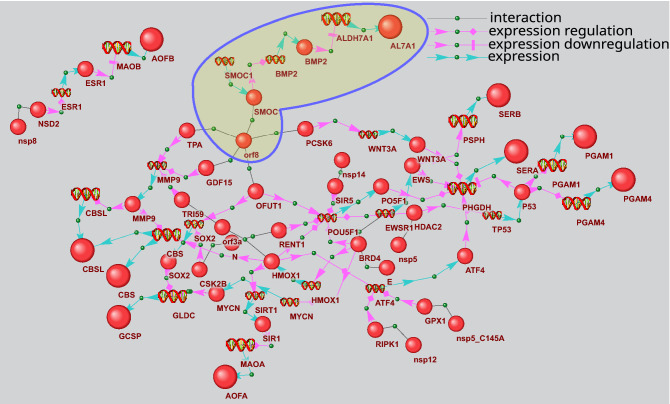
Gene network describing P_5_ pathways of gene expression regulation of glycine, serine and threonine metabolism. The bigger balls show the proteins of glycine, serine and threonine metabolism, while the smaller ones designate other proteins. Spirals designate the genes. The pathways discussed in the text are outlined.

Reconstruction of signaling pathways describing the potential regulation of glycine, serine and threonine metabolism is performed with the use of P_5_ template, which proves significant for this metabolic process (Table 4). P_5_ template describes the pathway of potential metabolic gene expression regulation by viral protein and two human intermediate genes/proteins. Of 40 genes of KEGG process of glycine, serine and threonine metabolism, ten are the potential targets of viral proteins (Supplementary Table S5). In Fig. 4, nine viral proteins participate in gene expression regulation of this metabolic process. Particularly, orf8 protein can influence the expression regulation of ALDH7A1 coding for AL7A1 protein. This enzyme (EC 1.2.1.8) participates in betaine biosynthesis (Fig. 3). The potential signaling pathway initiated by orf8 is represented by the following chain of interactions in gene network. Orf8 interacts with SMOC1^17^, SMOC1 can activate BMP2 gene expression^36^, while BMP2 inhibits ALDH7A1 expression^37^. As another example of signaling pathway from gene network shown in Fig. 4, we can consider the expression regulation of PHGDH gene coding for SERA (D-3-phosphoglycerate dehydrogenase) by viral protein nsp5. This enzyme (EC 1.1.1.95) catalyzes the reversible oxidation of 3-phospho-D-glycerate to 3-phosphonooxypyruvate, the first step of the phosphorylated L-serine biosynthesis pathway. According to the recent publication^17^, nsp5 interacts with histone deacetylase 2 (HDAC2). HDAC2 can, in turn, inhibit the expression of tumor suppressor p53^38–40^. The last link in nsp5-SERA signaling pathway is represented by p53 and PHGDH interaction. The former is known to suppress PHGDH (SERA) expression and inhibit serine biosynthesis^41^.

### Arginine biosynthesis

Arginine biosynthesis is the third top process among overrepresented KEGG processes (Table 3).

Of 23 metabolites participating in this process, six are significantly different between the plasma samples of COVID-19 patients and the controls (Supplementary Table S6). Five of them are increased in content, while ornithine is decreased (logFC =  − 1.98).

**Figure 5:**
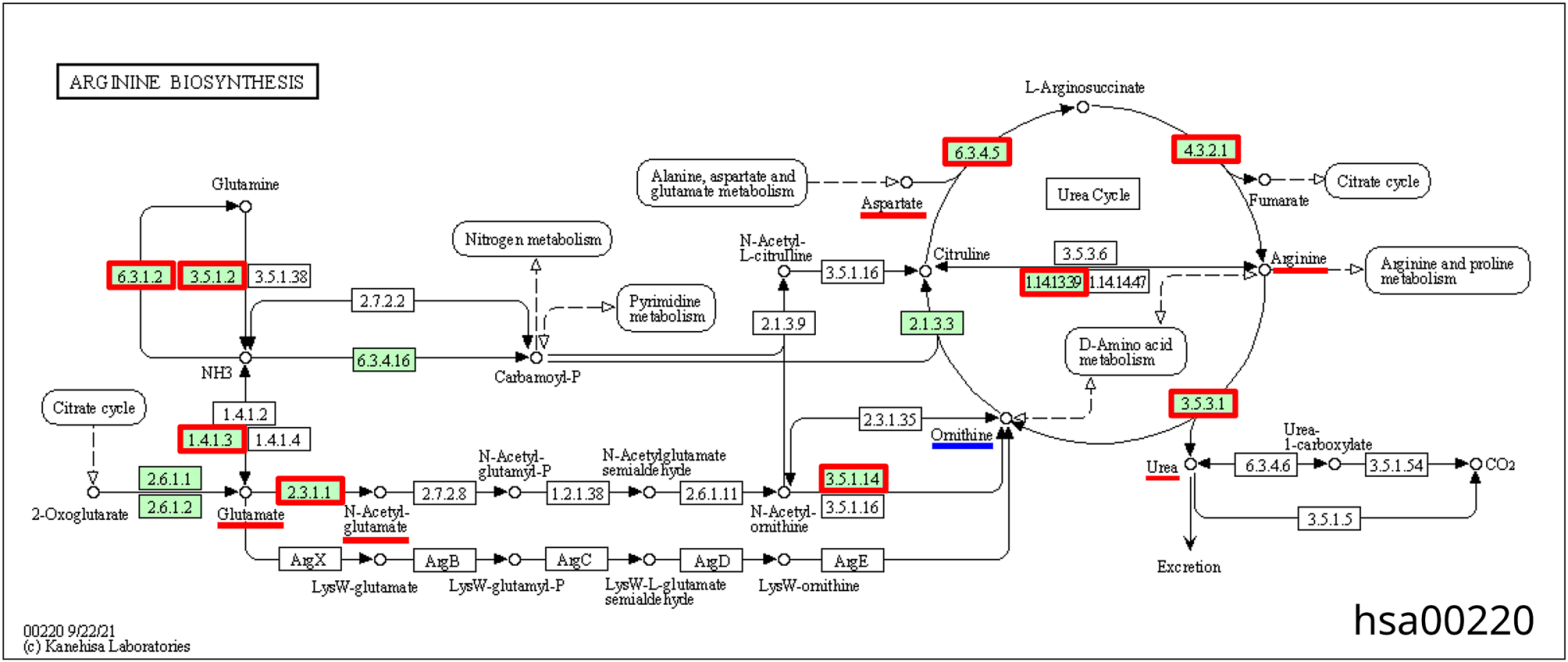
The scheme of arginine biosynthesis extracted from KEGG database^28^ (Id hsa00220). Enzymes that can be the potential targets of viral proteins are shown by red boxes, the metabolites increased in the plasma of COVID-19 patients are underscored by red line, while those decreased are underscored by blue line.

The potential regulation of arginine biosynthesis by viral proteins differs significantly from the two KEGG metabolic processes considered above (Supplementary Table S7). Of 22 genes involved in arginine biosynthesis, fourteen represent the potential targets of viral proteins (Fig. 5). Five types of signaling pathways potentially involved in the regulation prove statistically significant including pathways of P_2_, P_4_, P_5_, P_6_ and P_7_ types (Table 4). The viral proteins can potentially regulate the expression of enzymes of this metabolic process or the expression of human proteins, which interact with these enzymes, or they can control the activity or stability of these enzymes via the mentioned types of pathways.

**Figure 6:**
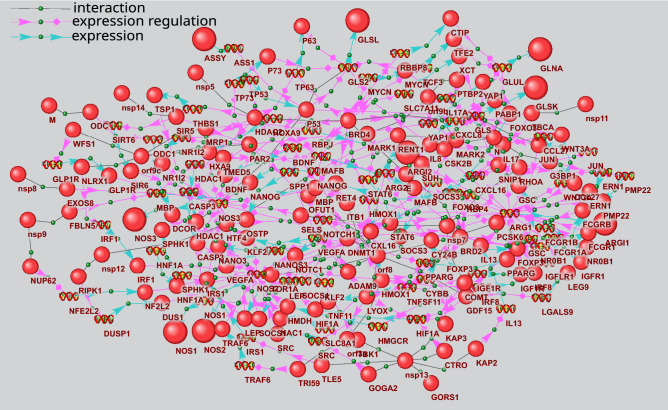
Gene network describing P_4_ and P_5_ pathways of arginine biosynthesis gene expression regulation. The bigger balls show the arginine biosynthesis proteins, while the smaller ones designate other proteins. Spirals designate the genes.

Gene network of expression regulation is shown in Fig. 6. It includes 74 genes and 132 proteins, nine of which are arginine biosynthesis enzymes, while 15 represent the viral proteins. The network also includes 194 links between proteins and genes, which describe expression regulation and 49 links defining the protein–protein interactions between viral and human counterparts.

**Figure 7:**
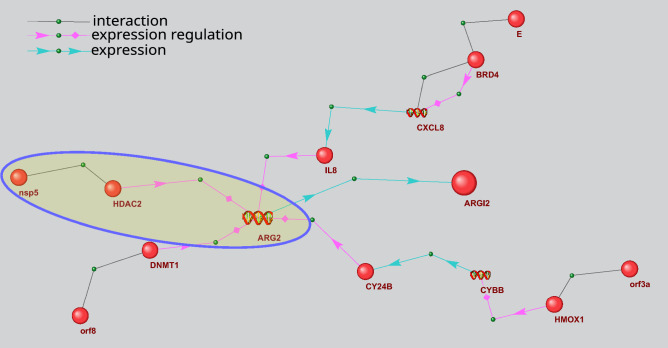
Pathways of arginase 2 (ARG2) expression regulation by viral proteins. The bigger ball designates ARG2, while the smaller ones designate other proteins. Spirals designate the genes. The pathways discussed in the text are outlined.

Figure 7 shows an example of signaling pathways included in gene network in Fig. 6, which can be involved in arginase 2 expression regulation. ARG2 (EC:3.5.3.1) participates in L-ornithine and urea synthesis from L-arginine and may play a role in the regulation of extra-urea cycle of arginine metabolism (Fig. 5). As seen in Fig. 7, viral proteins E, nsp5, orf8, and orf3a can regulate ARG2 expression. In particular, nsp5 can form complexes with histone deacetylase 2 (HDAC2) protein^17^. In turn, HDAC2 can suppress arginase 2 expression^42^. nsp5 binding to HDAC2 can potentially affect HDAC2 function. However, further experimental studies and computer simulations are needed to clarify the functional role of these protein–protein interactions. In particular, interesting results can be obtained by the computer-assisted structural modeling of protein complexes.

**Figure 8:**
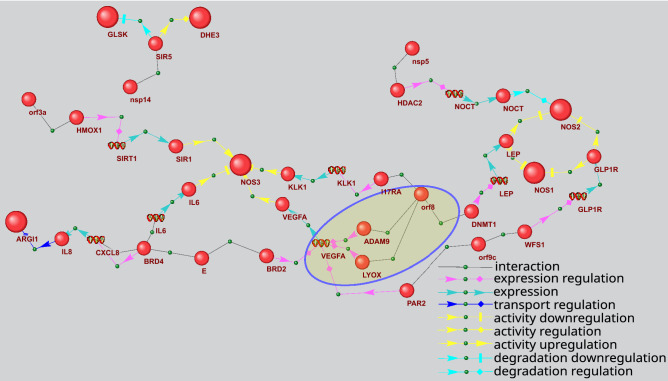
Gene network describing P_3_ and P_6_ pathways regulating activity/stability of arginine biosynthesis enzymes by viral proteins. The bigger balls show the proteins of arginine biosynthesis, while the smaller ones designate other proteins. Spirals designate the genes. The pathways discussed in the text are outlined.

An analysis of pathways related to the regulation of activity/stability of arginine biosynthesis enzymes reveals 6 viral proteins (E, nsp5, nsp14, orf3a, orf8 и orf9c) potentially involved in these pathways (Fig. 8). Six enzymes are associated with this type of regulation. NOS3 protein takes the central place in the network in this figure. Its activity can be potentially regulated by 4 viral proteins. Additionally, some of the latter can be involved in multiple ways, for example, orf8 and E participate in 3 and 2 pathways, respectively. Thus, one of the pathways involving orf8 is realized via its interaction with disintegrin and metalloproteinase domain-containing protein 9 (ADAM9), while another includes the interaction with protein-lysine 6-oxidase (LYOX). ADAM9 is known to increase vascular endothelial growth factor A (VEGFA) expression in lung cancer metastasis^43^. In the gene network, this interaction between ADAM9 and VEGFA is presented as a potential one. According to one publication^44^, LYOX positively regulates VEGFA expression. In turn, VEGFA can upregulate NOS3 function by phosphorylation of a specific serine residue^45^.

**Figure 9:**
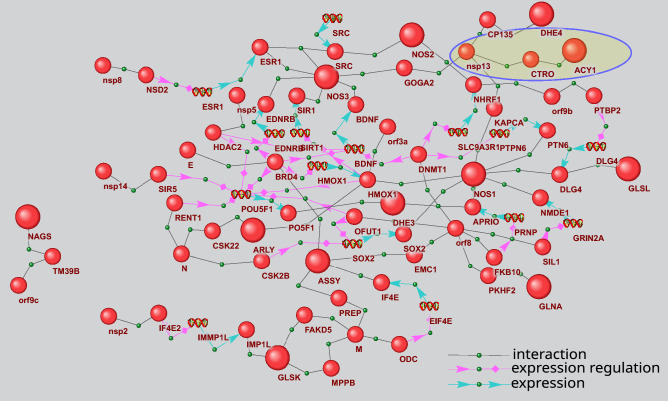
Gene network describing P_2_ and P_7_ pathways by which the viral proteins can influence the arginine biosynthesis proteins. The bigger balls show the proteins of arginine biosynthesis, while the smaller ones designate other proteins. Spirals designate the genes. The pathways discussed in the text are outlined.

Gene network presented in Fig. 9 describes the potential effects of viral proteins on protein–protein interactions of arginine biosynthesis enzymes. This network was reconstructed with the use of P_2_ and P_7_ templates. It includes 78 objects (15 genes and 63 proteins) and 94 interactions. Twelve viral proteins and twelve arginine biosynthesis enzymes participate in the network. P_2_ pathways describe the potential interactions of viral proteins with arginine biosynthesis enzymes, which are mediated by a single intermediate protein. For example, citron Rho-interacting kinase (CTRO) can mediate the interaction between nsp13 and aminoacylase-1 (ACY1), which is a part of arginine biosynthesis pathway. Protein–protein interactions between nsp13 and CTRO were described previously^17^, while CTRO interaction with ACY1 is included in BioGrid database (Id 2,538,152). One can expect that the first interaction (nsp13/CTRO) can have an adverse effect on the second one (CTRO/ACY1). However, the effect of such interaction on ACY1 function in arginine biosynthesis should be further studied. Interestingly, 4 proteins of arginine biosynthesis (NOS1, NOS3, DHE4 и ACY1) represent the potential nsp13 targets, which can be affected by this viral protein via P_2_ type pathways. The pathway linking viral protein E to NOS3 provides an example of P_7_ type pathways. It includes the following chain of potential interactions: E protein interacts with Bromodomain-containing protein 4 (BRD4) with the formation of a protein complex^17^, BRD4 regulates Endothelin receptor type B (EDNRB) gene expression^46^, while, according to HPRD database^47^, EDNRB interacts with NOS3 (HPRD Id 01,224, HPRD Id 01,224).

## Discussion and conclusions

Here, we report the results of metabolomic analysis of plasma samples of COVID-19 patients and the controls, which revealed 103 metabolites significantly differing between the two groups. According to the overrepresentation analysis of KEGG metabolic processes, the changes of metabolite content observed in the patients can be closely related to amino acid metabolism including arginine biosynthesis, glycine, serine and threonine metabolism, and aminoacyl-tRNA biosynthesis. The obtained results well correspond to the reported metabolomic analysis data of COVID-19 patient plasma samples^48–50^.

The metabolite most significantly differing between plasma samples of COVID-19 patients and the controls is mevalonolactone (Supplementary Table S1, Supplementary Note), a cyclic form of mevalonic acid, which plays a key role in cholesterol biosynthesis. Its content in plasma samples of COVID-19 patients was increased compared to that of the controls. The role of cholesterol biosynthesis in the mechanisms of COVID-19 pathology including the cytokine storm attracts keen attention of the researchers^27^. Additionally, the increased cholesterol content could be associated with the higher rate of infection due to the proposed capacity of the virus to use lipid rafts for cellular entry^51^.

Noteworthy, the previously published results of metabolomic studies were, typically, limited to the analysis of metabolic profiles and metabolic processes only. An interpretation of these data in terms of molecular mechanisms and roles of viral proteins in the revealed disturbed function of metabolic processes is often overlooked.

We used ANDSystem software, which enables one to reconstruct the gene networks from the data obtained by literature mining^18–20^, to analyze the potential role of SARS-CoV-2 proteins in the disturbed function of metabolic processes.

The potential contribution of viral proteins in the regulation of four metabolic processes discussed above is summarized in Supplementary Table S8. As seen from this table, most of the viral proteins can potentially be involved in the regulation of the reported metabolic pathways. Surprisingly, spike protein is not listed among the potential contributors, since the regulatory pathways, via which it could affect metabolic processes, were not revealed. Viral proteins E, N, nsp5, nsp8 and orf8 were the potential top contributors to the regulation of metabolic processes. Each of them was related to all four metabolic pathways reported above.

The results of analysis of aminoacyl-tRNA biosynthesis regulation pathways were rather interesting. First, we discovered that cytoplasmic and mitochondrial pathways of aminoacyl-tRNA biosynthesis regulation by viral proteins are different. Similar enzymes of the mitochondrial and cytoplasmic aminoacyl-tRNA biosynthesis are typically affected by different viral proteins. Also, the regulatory pathways via which the virus exerts its effects are different. Moreover, the reconstructed gene networks show that viral proteins do not affect the expression of genes coding for aminoacyl-tRNA biosynthesis enzymes, but rather influence the protein–protein interactions of these enzymes with other human proteins. Such interactions are known to affect protein activity and stability. This suggestion on the mechanisms of modulation of aminoacyl-tRNA biosynthesis by viral proteins well agrees with the results of publication by Huang and collaborators^11^, which focuses on the differential gene expression in SARS-CoV-2 infected pluripotent stem cell-derived human lung alveolar type 2 cells. This work demonstrates that LogFC of gene expression in the infected cells were not significantly higher or lower, than in non-infected cells (ranging from − 0.51 to 0.11).

Unlike the aminoacyl-tRNA biosynthesis, the two others overrepresented KEGG metabolic processes (glycine, serine and threonine metabolism and arginine biosynthesis) can be affected by viral proteins at the level of expression regulation of the genes coding for the enzymes involved in these pathways.

The regulation of glycine, serine and threonine metabolism can occur via the control over gene expression only. When analyzing the regulatory pathways of different types, we discovered that these were exactly the expression regulation pathways that proved statistically significant.

Of 40 genes of glycine, serine and threonine metabolism, ten represent the potential targets of viral proteins (Supplementary Table S8), and 9 viral proteins can be involved in the expression regulation of these genes.

The feature of the potential regulation of arginine biosynthesis by the viral proteins is that this process can be controlled via gene expression regulation, or the regulation of protein activity and stability, or the protein–protein interactions. Nine genes of this pathway show the expression regulation by viral proteins, while 7 and 13 proteins are regulated at the levels of activity/stability and protein–protein interactions, respectively.

Importantly, the reconstructed gene networks represent an initial attempt to the identification of molecular mechanisms of virus-host interactions in COVID-19 patients. The obtained results suggest the promising directions of research into the interactions between viral and host proteins. Our analysis shows that such interactions can initiate the transmission of regulatory signals along the chains of links between genes and proteins, which can enhance or inhibit gene expression or the activity of key enzymes.

According to our analysis of the reconstructed gene networks, the protein–protein interactions between viral and human proteins including orf8/GDF15, N/MOV10, nsp5/HDAC2 and others will have the most significant effect on the metabolic pathway functions.

The significance of the reconstruction of molecular mechanisms of pathogen-host interactions, including the approaches involving metabolomic analysis, is dictated by the serious problem of emerging drug resistance of viruses due to the high mutation rates of their genomes. The pharmacological influence over the functions of host genes, which are exploited by the virus for the benefit of its life cycle, may result in the development of new generation therapies^52^. One can expect that drug resistance to such therapies will be considerably lower, than to those drugs, which target the viral enzymes and genomes.

## Supplementary Information

Supplementary Information 1.

Supplementary Information 2.

Supplementary Information 3.

## Data Availability

All data generated or analysed during this study are included in this published article [and its supplementary information files].
