# Supplementary material for: Plasma metabolomics and gene regulatory networks analysis reveal the role of nonstructural SARS-CoV-2 viral proteins in metabolic dysregulation in COVID-19 patients
Source: Sci Rep. 2022 Nov 20;12:19977. doi: 10.1038/s41598-022-24170-0 (PMC9676188; doi:10.1038/s41598-022-24170-0)
Supplement: Supplementary file 2 — Supplementary Information 2. [file 41598_2022_24170_MOESM2_ESM.docx]

**Mevalonate pathway**

Mevalonolactone represents a cyclic form of mevalonic acid produced via condensation of the terminal hydroxyl and carboxyl groups, which remains in equilibrium with mevalonate in water. The conversion of hydroxymethylglutarylcoenzyme A (HMG-CoA) into mevalonate catalyzed by HMG-CoA reductase is the rate-limiting step of cholesterol biosynthesis. The lipid rafts, which represent the functional microdomains of the cell membrane enriched with cholesterol, play an important role in the early stages of Coronavirus Infectious Bronchitis Virus (IBN), especially, in the virus attachment to the cells mediated by the interaction of its structural proteins with the rafts [1]. Since mevalonolactone content is increased in plasma samples of the studied COVID-19 patient group, this metabolite can be considered as a potential diagnostic marker and a therapeutic target. Recently, the statins, which are HMG-CoA-reductase inhibitors, as well as some other lipid reducing drugs have been shown to represent a promising group of COVID-19 therapeutics, especially, for the patients with hearts and vessel disease and diabetes comorbidities [2].


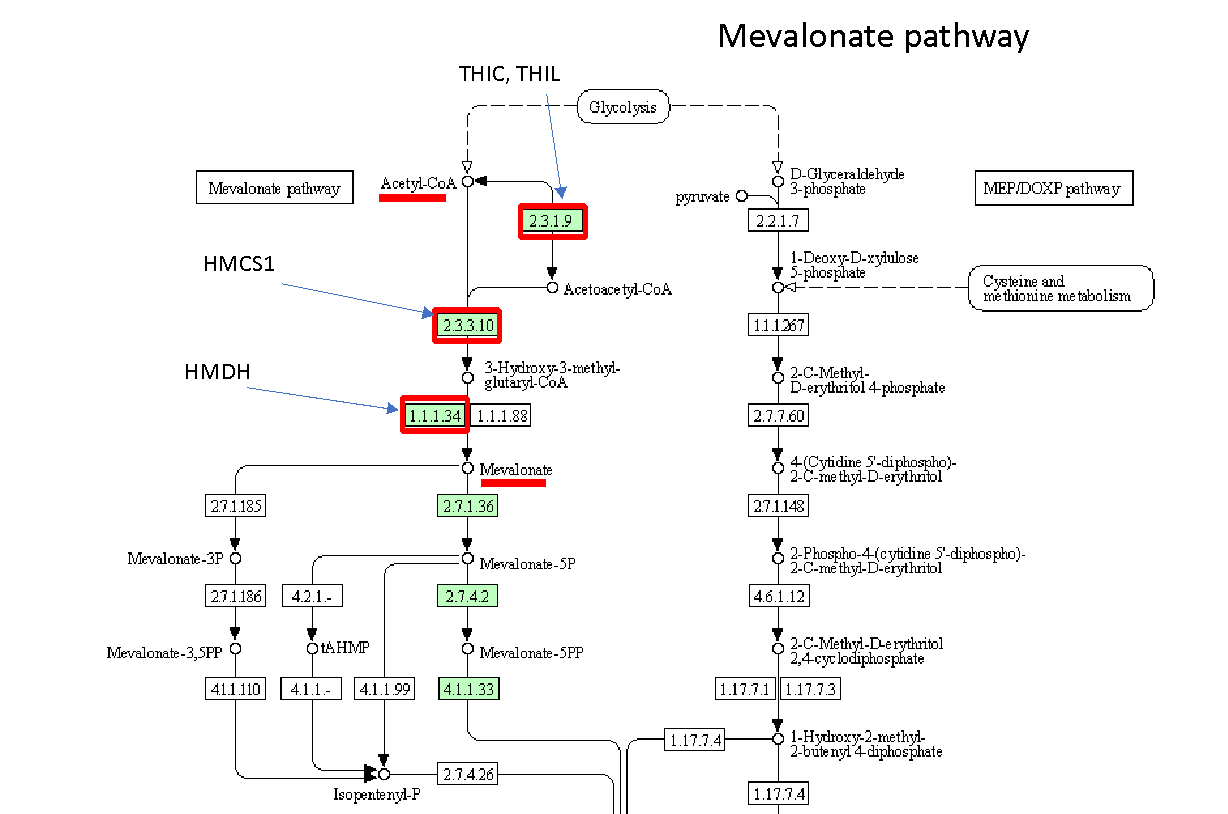


Figure S1. A fragment of KEGG terpenoid backbone biosynthesis process (Id hsa00900) including mevalonate production pathway [3]. Red boxes indicate the enzymes, which represent the potential viral protein targets.

Mevalonate production pathway is represented in KEGG as a part of terpenoid backbone biosynthesis process (hsa00900) (Fig. S1).

Table A1. Enzymes of Mevalonate production pathway, which are the potential targets of viral proteins according to the reconstruction of P_2_, P_5_, P_7_ types of signaling pathways.

| Gene symbol | Proteins | EC | Expression regulation,  templates (P_4_, P_5_) | Protein-protein interactions,  templates (P_2_, P_7_) |
| --- | --- | --- | --- | --- |
| ACAT2 | THIC | 2.3.1.9 |  | + |
| ACAT1 | THIL | 2.3.1.9 | + | + |
| HMGCR | HMDH | 1.1.1.34 | + | + |
| HMGS1 | HMGS1 | 2.3.3.10 | + |  |
| HMCS2 | HMCS2 | 2.3.3.10 |  | + |
|  |  |  |  |  |

The reconstruction of signaling pathway potentially involved in mevalonate pathway regulation by viral proteins revealed those related to expression regulation (for 3 enzymes) and the protein activity/stability regulation via protein-protein interactions with other human proteins (for 4 enzymes) (Tab. A1).

HMDH (3-hydroxy-3-methylglutaryl-coenzyme A reductase) is one of the key enzymes playing a critical role in cellular cholesterol homeostasis, which catalyzes the conversion of (3S)-hydroxy-3-methylglutaryl-CoA (HMG-CoA) to mevalonic acid, the rate-limiting step in the synthesis of cholesterol and other isoprenoids (Fig. S1). As recently published, HMDG gene shows an increased expression in SARS-CoV-2 infected alveolar epithelial type 2 cells [4].


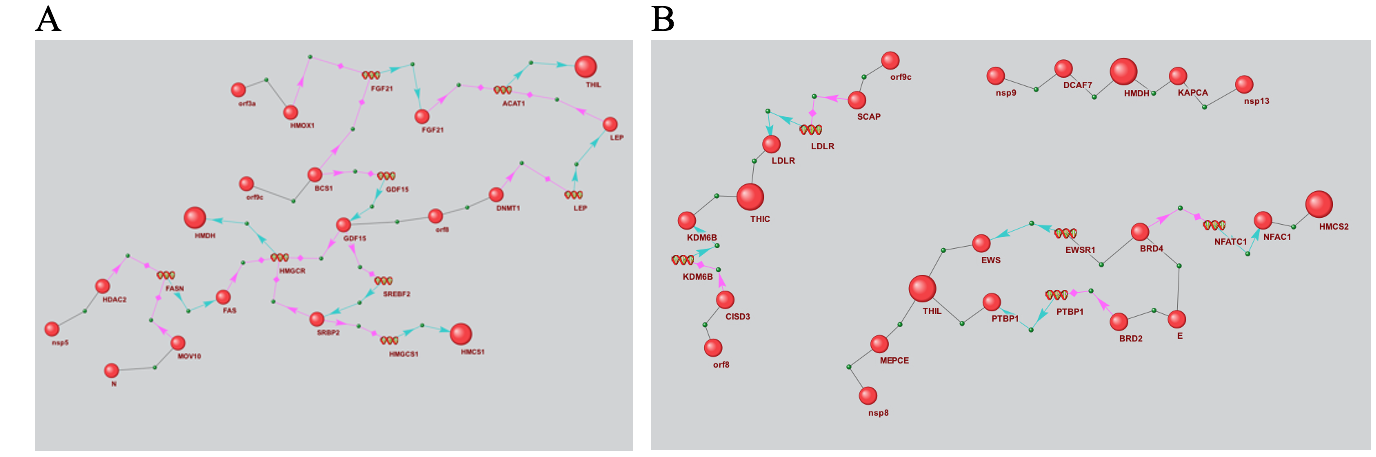


Figure S2. Gene networks describing the potential effects of viral proteins on mevalonate pathway proteins via expression regulation (A) and protein-protein interactions (B). Reconstruction of gene networks A and B was performed with the use of (P_4_, P_5_) and (P_2_, P_7_) templates, respectively. The bigger balls show the proteins of mevalonate pathway, while the smaller ones designate other proteins. Spirals designate the genes.

As seen in Fig. S2A, HMGCR gene coding for HMDG can be regulated by three proteins (SRBP2, CDF15 и FAS), which, in turn, can be influenced by the viral proteins. HMGRC expression can be activated by SREBP-2 [5, 6] and GDF15 [7] proteins. Meanwhile, GDF15 can also positively affect the expression of SREBF2 gene [7], while FAS acts as HMGCR expression inhibitor [8]. The effects of viral proteins on HMGCR expression can be described by the following pathways. Viral protein orf9c interacts with BCS1 protein, and this interaction can prevent the negative regulation of GDF15 expression by BCS1. Thus, orf9c exerts a positive effect on HMGCR expression. Viral protein orf9c can also directly bind to GDF15, however, the effect of this interaction on the GDF15 ability to positively regulate HMGCR and SREBF2 expression requires further investigation. Since HMGCR gene expression is enhanced in the infected cells, and taking into account our data on the increased mevalonate content in COVID-19 patient samples, one can expect that orf8 interaction with GDF15 will increase the potential of the latter to positively regulate HMGCR and SREBF2 expression. Viral proteins N and nsp5 can affect HMGCR expression via the regulation of MOV10 and HDAC2 proteins, which they can bind to. Meanwhile, both MOV10 and HDAC2 are the negative regulators of FASN expression [9, 10]. This implies that MOV10 and HDAC2 activity/stability should be increased by the interaction with the above viral proteins in order for them to have a positive effect on HMGCR expression.

An analysis of gene network shown in Fig. S2B enables us to propose the following hypotheses on the regulation of HMDG protein activity by the viral proteins. As seen in this picture, HMDG interacts with KAPCA (cAMP-dependent protein kinase catalytic subunit alpha). The information on this interaction is extracted from HPRD database [Id 00836] [11]. HMDH (3-hydroxy-3-methylglutaryl-coenzyme A reductase) is involved in protein-protein interactions with KAPCA (cAMP-dependent protein kinase catalytic subunit alpha). These interactions can modulate HMDG activity via the phosphorylation-dephosphorylation reaction [11]. HMDG phosphorylation is known to result in the loss of its activity [12]. In turn, KAPCA is affected by viral protein nsp13, which has an ability to interact with the former. The result of such protein-protein interaction on KAPCA activity remains unknown. If the interaction with nsp13 results in KAPCA inhibition and the loss of its ability to phosphorylate HMDG, this will positively regulate HMDG activity.

Fig. S2B reveals another partner of HMDG protein-protein interactions, the DCAF7 protein (DDB1- and CUL4-associated factor 7) (BioGrid Id 2539449). DCAF7 is involved in craniofacial development acting upstream of the EDN1 pathway, which is required for the formation of the upper jaw equivalent, the palatoquadrate. This protein is also related to the protein ubiquitination pathway. One can suggest that the interaction with DCAF7 results in HMDG ubiquitination and further degradation, similarly, as in case of DNA ligase I [13]. As seen in Fig. S2B, viral protein nsp9 interacts with DCAF7, as well. This interaction can also prevent DCAF7 interaction with HMDG and block the degradation of the latter. Thus, one of the possible functions of nsp9 and nsp13 can be an indirect positive effect on HMDG activity and stability.

**References**

1. Guo, H. et al. The Important Role of Lipid Raft-Mediated Attachment in the Infection of Cultured Cells by Coronavirus Infectious Bronchitis Virus Beaudette Strain. PLOS ONE vol. 12 e0170123 (2017).
2. Katsiki, N., Banach, M. & Mikhailidis, D. Lipid-lowering therapy and renin-angiotensin-aldosterone system inhibitors in the era of the COVID-19 pandemic. Archives of Medical Science vol. 16 485–489 (2020).
3. Kanehisa, M., Furumichi, M., Sato, Y., Ishiguro-Watanabe, M. & Tanabe, M. KEGG: integrating viruses and cellular organisms. Nucleic Acids Research vol. 49 D545-D551 (2021).
4. Huang, J. et al. SARS-CoV-2 Infection of Pluripotent Stem Cell-Derived Human Lung Alveolar Type 2 Cells Elicits a Rapid Epithelial-Intrinsic Inflammatory Response. Cell Stem Cell vol. 27 962-973.e7 (2020).
5. Howe, V., Sharpe, L. J., Prabhu, A. V. & Brown, A. J. New insights into cellular cholesterol acquisition: promoter analysis of human HMGCR and SQLE , two key control enzymes in cholesterol synthesis. Biochimica et Biophysica Acta (BBA) - Molecular and Cell Biology of Lipids vol. 1862 647–657 (2017).
6. Wong, T. Y., Lin, S. & Leung, L. K. The Flavone Luteolin Suppresses SREBP-2 Expression and Post-Translational Activation in Hepatic Cells. PLOS ONE vol. 10 e0135637 (2015).
7. Dong, G. et al. SCAP Mediated GDF15-Induced Invasion and EMT of Esophageal Cancer. Frontiers in Oncology vol. 10 (2020).
8. Che, L. et al. Cholesterol biosynthesis supports the growth of hepatocarcinoma lesions depleted of fatty acid synthase in mice and humans. Gut vol. 69 177–186 (2019).
9. Wang, W., Snyder, N., Worth, A. J., Blair, I. A. & Witze, E. S. Regulation of lipid synthesis by the RNA helicase Mov10 controls Wnt5a production. Oncogenesis vol. 4 e154–e154 (2015).
10. Feng, B. et al. Mitogen-Activated Protein Kinase Phosphatase 3 (MKP-3)–Deficient Mice Are Resistant to Diet-Induced Obesity. Diabetes vol. 63 2924–2934 (2014).
11. Prasad, T. S. K., Kandasamy, K. & Pandey, A. Human Protein Reference Database and Human Proteinpedia as Discovery Tools for Systems Biology. Methods in Molecular Biology 67–79 (2009) doi:10.1007/978-1-60761-232-2_6.
12. Beg, Z. H., Stonik, J. A. & Brewer, H. B., Jr. Characterization and regulation of reductase kinase, a protein kinase that modulates the enzymic activity of 3-hydroxy-3-methylglutaryl-coenzyme A reductase. Proceedings of the National Academy of Sciences vol. 76 4375–4379 (1979).
13. Peng, Z., Liao, Z., Matsumoto, Y., Yang, A. & Tomkinson, A. E. Human DNA Ligase I Interacts with and Is Targeted for Degradation by the DCAF7 Specificity Factor of the Cul4-DDB1 Ubiquitin Ligase Complex. Journal of Biological Chemistry vol. 291 21893–21902 (2016).
