# Supplementary material for: Plasma metabolomics and gene regulatory networks analysis reveal the role of nonstructural SARS-CoV-2 viral proteins in metabolic dysregulation in COVID-19 patients
Source: Sci Rep. 2022 Nov 20;12:19977. doi: 10.1038/s41598-022-24170-0 (PMC9676188; doi:10.1038/s41598-022-24170-0)
Supplement: Supplementary file 3 — Supplementary Information 3. [file 41598_2022_24170_MOESM3_ESM.docx]

Supplementary Table S1. The comparison of mean metabolite content in plasma samples of COVID-19 patients and the controls.

| Metabolite | logFC | p-value | Benjamini-Yekutieli test |
| --- | --- | --- | --- |
| mevalonolactone | 3,67385789 | 1,79E-48 | 1,38E-45 |
| inositol | 3,36129187 | 5,17E-29 | 3,63E-27 |
| L-Dihydroorotic acid_neg | 2,8402417 | 5,12E-44 | 1,98E-41 |
| 5-methoxytryptophan (2) | 3,2874407 | 1,74E-39 | 4,48E-37 |
| carnosine | 2,9773541 | 1,18E-31 | 1,30E-29 |
| aminoimidazole carboxamide ribonucleotide | 5,89364142 | 5,33E-22 | 2,29E-20 |
| imidazoleacetic acid | 2,93935153 | 3,23E-37 | 6,24E-35 |
| cystathionine | 2,97154212 | 2,41E-29 | 1,86E-27 |
| D-Sedoheptulose 7-phosphate (1)_neg | 2,06105489 | 1,93E-35 | 2,49E-33 |
| suberic acid | 1,95610554 | 7,85E-37 | 1,21E-34 |
| methionine sulfoxide | 2,50250136 | 2,37E-21 | 9,63E-20 |
| glucosamine | 2,27321 | 5,16E-30 | 4,44E-28 |
| L-Serine | 1,83756705 | 9,99E-25 | 4,83E-23 |
| homovanillic acid_neg | 2,06849072 | 9,42E-19 | 3,03E-17 |
| D-Glyceraldehyde 3-phosphate_neg | 1,75462919 | 1,32E-20 | 4,88E-19 |
| L-Homocysteic acid | 1,57049695 | 8,41E-27 | 4,64E-25 |
| Phosphorylcholine | 1,35051542 | 2,79E-27 | 1,66E-25 |
| pyridoxine | 1,91098555 | 1,58E-23 | 7,17E-22 |
| L-Alpha-aminobutyric acid | 1,11262311 | 1,96E-20 | 6,89E-19 |
| Orotic acid_neg | 1,28008137 | 2,10E-31 | 2,03E-29 |
| choline | 1,36280547 | 3,68E-21 | 1,42E-19 |
| L-Threonine | 1,24603543 | 3,86E-19 | 1,30E-17 |
| purine | 1,10541363 | 1,85E-18 | 5,71E-17 |
| aspartate | 2,02369946 | 1,58E-12 | 2,71E-11 |
| 5'-Methylthioadenosine | 1,48820443 | 1,18E-15 | 2,85E-14 |
| dimethylglycine | 0,90953551 | 2,18E-16 | 6,25E-15 |
| 4-Hydroxybenzoic acid_neg | 4,17016963 | 4,82E-09 | 5,91E-08 |
| Acetyllysine | 0,85645544 | 3,35E-12 | 5,28E-11 |
| taurine_neg | 1,44773494 | 8,00E-15 | 1,77E-13 |
| citric/Isocitric acid (2)_neg | -3,1513343 | 1,72E-12 | 2,89E-11 |
| IMP_neg | -1,9227111 | 8,43E-25 | 4,35E-23 |
| Indole-3-carboxylic acid_neg | -1,0510619 | 1,25E-28 | 8,07E-27 |
| Fumaric + maleic acid_neg | -2,096242 | 1,03E-12 | 1,82E-11 |
| L-Tyrosine | -1,0204155 | 3,57E-18 | 1,06E-16 |
| putrescine | -0,8712203 | 2,93E-11 | 4,05E-10 |
| Aminoadipic acid | -0,5714058 | 3,73E-16 | 9,95E-15 |
| p-coumaric acid | -0,9378339 | 5,40E-16 | 1,39E-14 |
| adenine | -0,7770586 | 1,73E-13 | 3,27E-12 |
| Alpha-ketoisovaleric acid_neg | -1,8379355 | 1,98E-11 | 2,84E-10 |
| Methylcysteine | -0,8127986 | 7,24E-14 | 1,40E-12 |
| 3-hydroxybutyric acid_neg | -2,7326292 | 2,03E-10 | 2,53E-09 |
| 2-Oxo-3-methyl-butyrate | -0,7221482 | 6,39E-14 | 1,27E-12 |
| Asymm + symm dimethylarginine | -0,7420297 | 1,22E-08 | 1,46E-07 |
| L-Leucine + L-isoleucine | -0,5929174 | 2,01E-12 | 3,30E-11 |
| uridine_neg | -0,7372185 | 5,87E-12 | 8,90E-11 |
| 2-hydroxybutanoic acid_neg | -0,986519 | 4,76E-08 | 5,42E-07 |
| asparagine | -1,6101811 | 5,07E-11 | 6,65E-10 |
| Glyceric acid_neg | -0,6794483 | 4,28E-07 | 4,60E-06 |
| dUMP_neg | -0,6946487 | 2,39E-07 | 2,61E-06 |
| ornithine | -1,9805433 | 0,000462216 | 4,02E-03 |
| 7-methylguanosine | -0,4551247 | 0,000145994 | 1,34E-03 |
| Uric acid_neg | -0,5707188 | 0,016504543 | 1,19E-01 |
| Indoleacrylic acid_neg | 0,72236588 | 0,041618891 | 2,90E-01 |
| L-Methionine | -0,5902028 | 5,47E-05 | 5,22E-04 |
| Citraconic acid_neg | -0,2124714 | 0,008108529 | 6,03E-02 |
| trimethylamine oxide | 0,04883983 | 0,370766265 | 1,00E+00 |
| N-Acetyl-L-alanine_neg | -0,1070064 | 0,131921224 | 8,43E-01 |
| Allantoic acid_neg | -0,2998394 | 0,000607162 | 5,10E-03 |
| pantothenic acid | -0,3636226 | 0,006067168 | 4,55E-02 |
| Tetradecanedioic acid_neg | -0,4072635 | 0,00103227 | 8,31E-03 |
| Phenyllactic acid (2)_neg | -1,7684531 | 1,35E-06 | 1,38E-05 |
| L-Glutamine (?) | -0,251761 | 0,069917324 | 4,62E-01 |
| hippuric acid_neg | -0,099896 | 0,24440064 | 1,00E+00 |
| hippuric acid | -0,1101483 | 0,224783414 | 1,00E+00 |
| gluconic acid_neg | 0,00500137 | 0,535958194 | 1,00E+00 |
| Pantothenic acid_neg | -0,2389065 | 0,021043279 | 1,51E-01 |
| carnitine | -0,0979149 | 0,175375363 | 1,00E+00 |
| Ureidosuccinic acid_neg | -0,1651364 | 0,052688255 | 3,54E-01 |
| citrulline | 0,11378014 | 0,850619174 | 1,00E+00 |
| glucose_neg | -0,0420888 | 0,406494602 | 1,00E+00 |
| L-Tryptophan | -0,0918018 | 0,571516923 | 1,00E+00 |
| Acetylcarnitine | -0,0533872 | 0,248619074 | 1,00E+00 |
| Alpha-N-Phenylacetyl-L-glutamine | 0,47865444 | 0,135789286 | 8,61E-01 |
| L-Histidine | -1,9327941 | 0,69377864 | 1,00E+00 |
| d-glucose_neg | 0,07955135 | 0,530298534 | 1,00E+00 |
| Hydroxyisocaproic acid_neg | -0,0793304 | 0,438623484 | 1,00E+00 |
| Quinolinic acid | -0,4155716 | 0,764396906 | 1,00E+00 |
| Kynurenine (1)_neg | 0,14111656 | 0,356073355 | 1,00E+00 |
| Pyridoxamine | 0,11766081 | 0,349358565 | 1,00E+00 |
| Melatonin | 0,29035062 | 0,046806637 | 3,20E-01 |
| Cystine (1) | 0,14817648 | 0,382019843 | 1,00E+00 |
| Homogentisic acid_neg | 0,10264779 | 0,753872293 | 1,00E+00 |
| acetyl-CoA (2) | 0,21852623 | 0,043499818 | 3,00E-01 |
| caffeine | 0,93245647 | 0,058675828 | 3,91E-01 |
| L-Kynurenine (2) | 0,24907237 | 0,02125451 | 1,51E-01 |
| xanthine | 0,47394602 | 0,185858078 | 1,00E+00 |
| Pyroglutamic acid_neg | 0,39958076 | 0,000427558 | 3,76E-03 |
| cytidine | 0,53397963 | 0,008791238 | 6,47E-02 |
| 2-Aminooctanoic acid (1) | 0,46452421 | 0,014118831 | 1,03E-01 |
| L-Valine | 0,19124677 | 0,003487071 | 2,67E-02 |
| serotonin | 0,34636115 | 0,004114122 | 3,12E-02 |
| Guanidinoacetic acid | 0,58737217 | 0,002572407 | 2,01E-02 |
| Lipoamide | 0,26804127 | 0,022020972 | 1,55E-01 |
| Methylmalonic acid_neg | -0,0831625 | 0,112853779 | 7,33E-01 |
| Pyroglutamic acid | 0,82149211 | 0,00135083 | 1,07E-02 |
| xanthosine | 1,09735919 | 0,0012806 | 1,02E-02 |
| L-Phenylalanine | 0,38755982 | 1,25E-06 | 1,28E-05 |
| L-Proline | 0,50615208 | 1,89E-05 | 1,85E-04 |
| Ascorbic acid | 0,60627981 | 9,96E-05 | 9,28E-04 |
| creatine | 0,86791956 | 0,001013437 | 8,25E-03 |
| cystine (2) | 0,32717523 | 0,12219803 | 7,87E-01 |
| Urea | 0,39414496 | 0,000519712 | 4,42E-03 |
| L-Kynurenine (1) | 0,4242691 | 0,000336965 | 2,99E-03 |
| N-acetyl-glutamine | 0,49453193 | 0,000146798 | 1,34E-03 |
| Succinic acid_neg | -0,0380453 | 0,052146044 | 3,54E-01 |
| Lysine + glutamine | 0,50176552 | 3,87E-05 | 3,74E-04 |
| mevalonic acid | 0,48925322 | 3,49E-08 | 4,03E-07 |
| Glycerophosphocholine | 0,65839245 | 0,000155815 | 1,40E-03 |
| indole | 0,43943169 | 0,000955645 | 7,90E-03 |
| uracil | 0,30812023 | 0,000960748 | 7,90E-03 |
| 3-hydroxykynurenine | 0,06408795 | 0,081261522 | 5,32E-01 |
| 1,4-diaminobutane | 0,4314099 | 0,000472526 | 4,06E-03 |
| beta aminobutyric acid | 0,82552436 | 2,90E-13 | 5,34E-12 |
| L-Arginine (1) | 1,52940493 | 3,00E-06 | 3,01E-05 |
| L-Homoserine | 0,80096086 | 6,26E-07 | 6,63E-06 |
| 5-Hydroxy-L-tryptophan | 0,82043871 | 4,94E-08 | 5,54E-07 |
| Inosine | 2,67136568 | 9,48E-08 | 1,05E-06 |
| betaine | 0,33185671 | 8,93E-05 | 8,42E-04 |
| L-Lactic acid_neg | 0,39626744 | 7,29E-06 | 7,23E-05 |
| 3-Aminoisobutanoic acid | 0,680343 | 2,95E-12 | 4,75E-11 |
| Aminoadipic acid_1 | 1,25493025 | 2,71E-15 | 6,34E-14 |
| Gamma-Aminobutyric acid | 0,86616712 | 1,53E-14 | 3,29E-13 |
| Glutamate | 1,73743227 | 2,65E-14 | 5,39E-13 |
| riboflavin | 0,85160135 | 3,44E-11 | 4,58E-10 |
| 1-Methylnicotinamide | 2,53249239 | 6,66E-16 | 1,66E-14 |
| hypoxanthine | 2,51668597 | 3,49E-16 | 9,64E-15 |
| 1-Methyladenosine | 1,23656137 | 6,77E-12 | 1,01E-10 |
| creatinine | 0,88029704 | 1,95E-10 | 2,47E-09 |
| Deoxyribose 1-phosphate_neg | 0,70673593 | 4,80E-15 | 1,09E-13 |
| D-glucuronic acid | 1,99000492 | 7,39E-12 | 1,08E-10 |
| L-Alanine | 0,80774137 | 5,21E-12 | 8,05E-11 |
| Pipecolic acid | 1,28647754 | 9,32E-13 | 1,68E-11 |
| sarcosine | 0,82584195 | 3,17E-11 | 4,30E-10 |
| N-Acetylglutamic acid (1)_neg | 1,62000821 | 2,02E-14 | 4,22E-13 |
| Imidazole | 0,30398296 | 7,02E-11 | 9,05E-10 |
| methylsuccinic acid | 1,55760318 | 1,37E-08 | 1,61E-07 |
| Metanephrine | 0,95040278 | 7,44E-07 | 7,78E-06 |
| palmitic acid_neg | 0,76648318 | 2,21E-11 | 3,11E-10 |
| histamine | -0,7228762 | 0,00316354 | 2,45E-02 |
| AICA-riboside | 0,51063267 | 6,40E-09 | 7,73E-08 |

Supplementary Table S2. Metabolites of aminoacyl-tRNA biosynthesis process, the content of which significantly differed between blood plasma samples of COVID-19 patients and the controls.

| Metabolite | KEGG Id | logFC | Benjamini-Yekutieli test |
| --- | --- | --- | --- |
| L-Asparagine | C00152 | -1.61 | 6,6E-10 |
| L-Phenylalanine | C00079 | 0.38 | 1,2E-05 |
| L-Arginine | C00062 | 1.52 | 3E-05 |
| L-Aspartic acid | C00049 | 2.02 | 2,7E-11 |
| L-Serine | C00065 | 1.83 | 4,828E-23 |
| L-Methionine | C00073 | -0.59 | 5,2E-04 |
| L-Valine | C00183 | 0.19 | 2,6E-02 |
| L-Alanine | C00041 | 0.8 | 8E-11 |
| L-Lysine | C00047 | 0,5 | 3,7E-04 |
| L-Leucine | C00123 | -0.59 | 3,2E-11 |
| L-Threonine | C00188 | 1.24 | 1,3E-17 |
| L-Tyrosine | C00082 | -1.02 | 1,06E-16 |
| L-Proline | C00148 | 0.5 | 1,8E-04 |
| L-Glutamic acid | C00025 | 1.73 | 5,3E-13 |

Supplementary Table S3. Enzymes of aminoacyl-tRNA biosynthesis process, which are the potential targets of viral proteins according to the reconstruction of P_2_ and P_7_ types of signaling pathways.

| EC number | Mitochondrial | | Virus regulation | | Cytoplasmic | | Virus regulation | |
| --- | --- | --- | --- | --- | --- | --- | --- | --- |
|  | Gene simbol | Protein | Template P_2_ | Template P_7_ | Gene simbol | Protein | Template P_2_ | Template P_7_ |
| 6.1.1.22 | NARS2 | SYNM | - | + | MARS1 | SYNC | + | - |
| 6.1.1.20 |  | - | - | - | FARSB | SYFA, SYFB | + | + |
| 6.1.1.19 | RARS2 | SYRM | + | + | RARS1 | SYRC | + | + |
| 6.1.1.12 | DARS2 | SYDM | + | - | DARS1 | SYDC | - | - |
| 6.1.1.11 | SARS2 | SYSM | - | + |  | - | - | - |
| 2.1.2.9/6.1.1.10 | MTFMT | FMT | + | + | MARS1 | SYMC | + | + |
| 6.1.1.9 | VARS2 | SYVM | + | + |  | - | - | - |
| 6.1.1.7 | AARS2 | SYAM | + | + | AARS1 | SYAC | + | + |
| 6.1.1.4 | LARS2 | SYLM | + | + | LARS1 | SYLC | + | + |
| 6.1.1.5 | IARS2 | SYIM | + | + |  | - | - | - |
| 6.1.1.3 |  | - | - | - | TARS1 | SYTC, SYTC2 | + | + |
| 6.1.1.17 |  | - | - | - | EPRS1 | SYEP | + | + |
| 6.3.5.7/6.1.1.17 | QRSL1 | GATA | + | - | EPRS1 | SYEP | + | + |

Supplementary Table S4. Metabolites of KEGG process of glycine, serine and threonine metabolism, which were significantly differing in content between the COVID-19 patient and the control plasma samples.

| metabolite | logFC | Benjamini-Yekutieli test |
| --- | --- | --- |
| L-Serine | 1,83 | 4,82E-23 |
| Choline | 1,36 | 1,42E-19 |
| Betaine | 0,33 | 0,00084 |
| Guanidoacetic acid  Guanidinoacetic acid | 0,58 | 2,0E-02 |
| Dimethylglycine | 0,9 | 6,24E-15 |
| L-Cystathionine | 2,97 | 1,86E-27 |
| Sarcosine | 0,82 | 4,3E-10 |
| L-Threonine | 1,24 | 1,29E-17 |
| Glyceric acid | -0,67 | 4,6E-06 |
| Creatine | 0,86 | 8,2E-03 |

Supplementary Table S5. Genes involved in metabolic process of glycine, serine and threonine metabolism, the expression of which can be potentially regulated by viral proteins according to the reconstruction of P_5_ signaling pathways.

| Gene symbol | Proteins | EC | FDR | LogFC |
| --- | --- | --- | --- | --- |
| ALDH7A1 | AL7A1 | 1.2.1.8 | 1,2e-07 | -0,85 |
| MAOA | AOFA | 1.4.3.4 | 0,01 | -0,2 |
| MAOB | AOFB | 1.4.3.4 | - | - |
| CBS | CBS | 4.2.1.22 | 0.02 | 0.38 |
| CBSL | CBSL | 4.2.1.22 | 0.09 | 0.43 |
| GLDC | GCSP | 1.4.4.2 | 0,11 | 0,24 |
| PGAM1 | PGAM1 | 5.4.2.11 | 0.047 | -0,42 |
| PGAM4 | PGAM4 | 5.4.2.11 | - | - |
| PHGDH | SERA | 1.1.1.95 | 0,0033 | -0,48 |
| PSPH | SERB | 3.1.3.3 | 0,0027 | -0,47 |

Supplementary Table S6. Metabolites of KEGG process of arginine biosynthesis, which were significantly differing in content between the COVID-19 patient and the control plasma samples.

| Metabolite | KEGG Id | logFC | Benjamini-Yekutieli test |
| --- | --- | --- | --- |
| L-Glutamic acid | C00025 | 1,73 | 5,39E-13 |
| L-Arginine | C00062 | 1,52 | 3,01E-05 |
| L-Aspartic acid | C00049 | 2,02 | 2,71E-11 |
| Ornithine | C00077 | -1,98 | 4,01E-03 |
| N-Acetylglutamic acid | C00624 | 1,62 | 4,22E-13 |
| Urea | C00086 | 0,39 | 4,41E-03 |

Supplementary Table S7. Genes involved in the metabolic process of arginine biosynthesis, the expression of which can be potentially regulated by viral proteins according to the reconstruction of signaling pathways.

| Genes simbol | Proteins | EC | FDR | LogFC | Expression regulation (templates P_4_, P_5_) | Activity/stability regulation (P_6_ template) | Protein-protein interactions (templates P_2_, P_7_) |
| --- | --- | --- | --- | --- | --- | --- | --- |
| **NOS3** | **NOS3** | **1.14.13.39** | **4,3E-06** | **1,47** | **+** | **+** | **+** |
| ASS1 | ASSY | 6.3.4.5 | 0,026 | 1,39 | + | + | + |
| **ARG2** | **ARGI2** | **3.5.3.1** | **4,03E-05** | **1,22** | **+** |  |  |
| GLS | GLSK | 3.5.1.2 | 5,5E-06 | 0.52 | + | + | + |
| GLUD2 | DHE4 | 1.4.1.3 | 0.31 | -0.1 |  |  | + |
| GLUD1 | DHE3 | 1.4.1.3 | 0.0003 | -0.27 |  | + | + |
| ASL | ARLY | 4.3.2.1 | 0.0003 | -0.62 |  |  | + |
| ACY1 | ACY1 | 3.5.1.14 | 0.0027 | -0.57 |  |  | + |
| GLUL | GLNA | 6.3.1.2 | 0.0017 | -0.24 | + |  | + |
| NAGS | NAGS | 2.3.1.1 | 0.044 | -0.53 |  |  | + |
| NOS2 | NOS2 | 1.14.13.39 | - | - | + | + | + |
| NOS1 | NOS1 | 1.14.13.39 | - | - | + | + | + |
| GLS2 | GLSL | 3.5.1.2 | 0.74 | -0.06 | + |  | + |
| ARG1 | ARGI1 | 3.5.3.1 | 0.19 | 0.47 | + | + |  |

Supplementary Table S8. Potential contribution of viral proteins to the regulation of metabolic processes, as revealed by gene network reconstruction.

| SARS-CoV-2 proteins | Aminoacyl-tRNA biosynthesis | Glycine, serine and threonine metabolism | Arginine biosynthesis | Melavonate pathway |
| --- | --- | --- | --- | --- |
| E | + | + | + | + |
| N | + | + | + | + |
| nsp5 | + | + | + | + |
| nsp8 | + | + | + | + |
| orf8 | + | + | + | + |
| orf3a |  | + | + | + |
| orf9c | + |  | + | + |
| nsp12 |  | + | + |  |
| nsp13 |  |  | + | + |
| nsp14 |  | + | + |  |
| nsp2 | + |  | + |  |
| nsp9 |  |  | + | + |
| nsp5_C145A | + | + |  |  |
| M | + |  | + |  |
| nsp10 | + |  |  |  |
| nsp11 |  |  | + |  |
| nsp7 |  |  | + |  |
| orf9b |  |  | + |  |
| orf10 | + |  |  |  |
| Spike |  |  |  |  |
| nsp1 |  |  |  |  |
| nsp4 |  |  |  |  |
| nsp6 |  |  |  |  |
| nsp15 |  |  |  |  |
| orf3b |  |  |  |  |
| orf6 |  |  |  |  |
| orf7a |  |  |  |  |
